# Surgical selection and regional lymph node dissection for stage I second primary lung cancer patients following surgery for stage I first primary lung cancer

**DOI:** 10.3389/fonc.2023.1148422

**Published:** 2023-03-28

**Authors:** Xiao Wu, Youhua Jiang, Qixun Chen, Jiangfeng Wang, Jianqiang Li

**Affiliations:** Department of Thoracic Surgery, The Cancer Hospital of the University of Chinese Academy of Sciences (Zhejiang Cancer Hospital), Hangzhou, China

**Keywords:** second primary lung cancer, SEER, lung cancer-specific mortality, lymph node dissection, surgery

## Abstract

**Introduction:**

Studies investigating surgery for second primary non-small cell lung cancer (SP) patients are rare. The aim of this study was to explore the effects of surgical methods and regional lymph node (LN) dissection on lung cancer-specific mortality (LCSM) in stage I SP patients following surgery for stage I first primary non-small cell lung cancer (FP).

**Methods:**

Data on patients diagnosed with stage I SP after surgery for stage I FP were extracted from the Surveillance, Epidemiology, and End Results (SEER) database. Cumulative incidence function (CIF) curves, a competing risk model and propensity score matching (PSM) were adopted to compare the LCSM among different subgroups (including surgery and regional LN dissection).

**Results:**

A total of 238 stage I SP patients were extracted from the SEER database. Overall, the 5-year LCSM rate was 29.8% (CI: 23.1%-36.5%) for the whole cohort. Both before and after PSM, lobectomy had a similar LCSM incidence as sublobectomy, and ≥4 regional LN dissections had a significantly lower LCSM incidence than 1~3 regional LN dissections.In addition, patients who underwent 1~3 regional LN dissections had a comparable incidence of LCSM to those without LN dissections.

**Discussion:**

Stage I SP patients tended to gain more survival benefits when surgeons dissect ≥4 regional LNs. Allowing for the comparable LCSM incidence of sublobectomy to lobectomy, sublobectomy may be a reasonable choice for thoracic surgeons when performing surgery for these patients.

## Introduction

Lung cancer is the second most commonly diagnosed cancer in the United States (US) and is the leading cause of malignancy-related mortality (1). Currently, as low-dose helical computed tomography is prevalently adopted in the clinic, an increasing number of stage I non-small cell lung cancer (NSCLC) patients are being identified (2). For these patients, surgical treatment is still perceived as the best treatment option (3), and the 5-year survival rates are 90%, 85%, 80%, and 73% for pathologic stages IA1, IA2, IA3, and IB, respectively (4). A better survival, however, denotes that stage I NSCLC patients have more time to progress to a second primary lung cancer (5–7). Therefore, it is imperative to investigate the surgical information for second primary non-small cell lung cancer (SP) patients following the diagnosis of stage I first primary non-small cell lung cancer (FP).

To our knowledge, several studies have explored surgical treatment for SP patients (5, 6, 8–11). However, it is still controversial whether lobectomy is superior to sublobectomy in stage I SP. Several studies found no survival difference between sublobectomy and lobectomy (12–14), while others revealed that lobectomy was associated with better survival than sublobectomy (15, 16). In addition, it was discovered that more extensive regional lymph node (LN) dissection is related to better survival in stage I SP. However, these studies did not adopt a competing risk model, which inevitably overestimated the incidence of lung cancer-specific mortality (LCSM) (17, 18). As noted, previous studies seldom took the pathologic staging of FP into consideration (13), and this factor can significantly influence the survival of stage I SP patients. In addition, as treatment information (such as chemotherapy, targeted therapy, or immunotherapy) was not available in the SEER database, stage II, III, IV patients generally should receive the above adjuvant treatment after surgery (3). Therefore, it is not reasonable to incorporate stage II, III, IV patients into the study. From this perspective, we confined our study population to stage I patients (both FP and SP), which could eliminate the interference from non-surgical treatment.

Thus, the main aim of this study was to investigate the effect of surgical treatment and the scope of regional LN dissection on LCSM in stage I SP patients following postoperative stage I FP.

## Materials and methods

### Data sources

**Figure 1:**
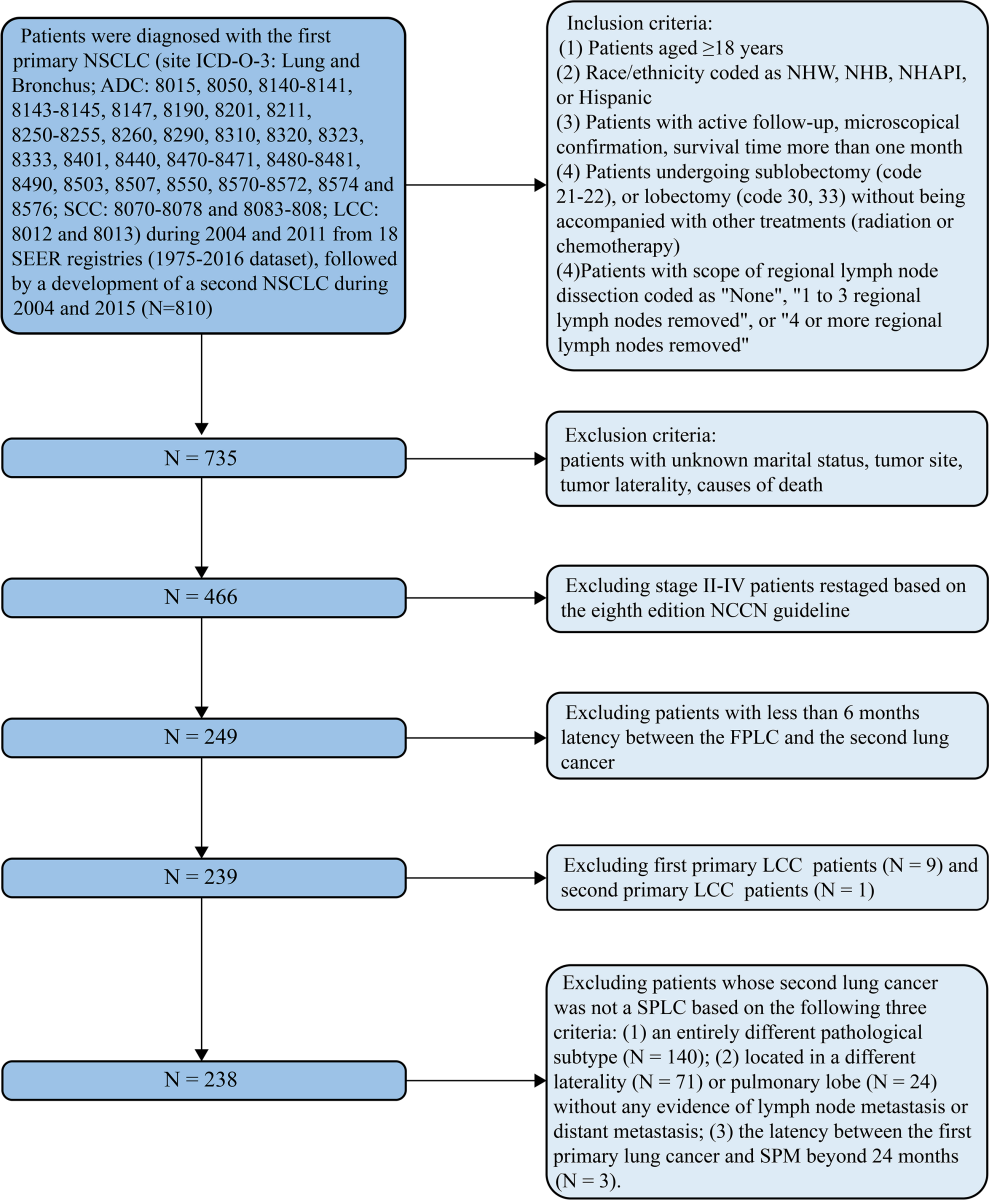
Flow diagram described the screening process in the SEER database, containing the inclusion and exclusion criteria. ADC, adenocarcinoma; LCC, large cell carcinoma; FP, first primary lung cancer; NCCN, National Comprehensive Cancer Network; NHAPI, non-Hispanic Asian/Pacific Islander; NHB, non-Hispanic Black; NHW, non-Hispanic white; NSCLC, non-small cell lung cancer; SCC, squamous cell cancer; SEER, Surveillance Epidemiology and End Results; SP, second primary non-small cell lung cancer.

Patients aged 18 years and above who were diagnosed with stage I first primary non-small cell lung cancer (FP) between January 2004 and December 2011 and had undergone surgical treatment without radiation or chemotherapy were extracted from the Surveillance, Epidemiology, and End Results (SEER) database (1975–2016 dataset). Among them, we further extracted those patients who subsequently developed a second stage I primary non-small cell lung cancer (SP) from January 2004 to December 2015 and once again underwent surgery without radiation or chemotherapy. Since the development of an SPM was closely related to the length of follow-up, we set the cutoff diagnostic time point of the FP at the year 2011, which ensured a minimal 5-year follow-up period. Moreover, we set the cutoff diagnostic time point of the SP at the year 2015, which ensured a minimal 1-year follow-up period. As the SEER database is lacking in treatment data (such as chemotherapy regimens, immunotherapy, and targeted therapy) and these treatments are essential for middle-stage and late-stage non-small cell lung cancer (NSCLC) patients, we did not investigate these patients and confined our study population to stage I NSCLC patients. Surgery was classified into two categories based on the surgical codes: sublobectomy (code 21 or 22) and lobectomy (codes 30 or 33). The eighth edition of the American Joint Committee on Cancer (AJCC) TNM staging system was adopted to restage all the included patients, and stage I was defined as T1-T2aN0M0 (3). The scope of regional LN dissection was classified into three categories: “none”, “1 to 3 regional LNs removed”, or “4 or more regional LNs removed”. These three items were also the default categories in the SEER database. It is generally believed that adenocarcinoma, squamous cell carcinoma (SCC), and large cell carcinoma are the three most common histological subtypes of NSCLC (19). As only one patient was diagnosed with stage I second primary large cell lung cancer, we omitted this subtype and focused on adenocarcinoma and SCC only. A flow chart that contained the inclusion and exclusion criteria is displayed in **Figure 1**.

### Identification of an SPM

Two important variables in the SEER database, “sequence number” and “total number of in situ/malignant tumors or patient”, were utilized to identify the SPM. The former was used to determine the sequence of multiple primary malignancies, while the latter was used to distinguish SPM patients. Whether a second primary lung cancer can be perceived as an SPM depends on the following three criteria (20): (1) an entirely different pathological subtype from the first primary lung cancer; (2) the latency between the first primary lung cancer and SPM beyond 24 months; and (3) tumor located in a different pulmonary lobe, without any evidence of LN metastasis or distant metastasis (**Figure 1**).

### Statistical analysis

The significant differences in clinicopathological variables between the different groups were assessed using Wilcoxon-Wilcox tests for continuous variables without normal distribution and chi-squared tests (with or without continuity correction) for categorical variables. We used the cumulative incidence function (CIF) to estimate the cumulative incidences of LCSM, and the differences in CIF were evaluated using Gray’s test (21).

In this study, death from lung cancer was defined as lung cancer-specific mortality (LCSM) and regarded as a main event, while death from other cause-specific morality (OCSM) was perceived as a competing risk event. Variables that were considered clinically related beforehand or had significant differences in the univariate competing risk model (P<0.05) were included in the subsequent multiple stepwise regression model. Ultimately, the best multivariate competing risk model was fitted, and subdistribution hazard ratios (SHRs) of the included variables were obtained from the model. In addition, we used propensity score matching (PSM) to compare the survival disparities between the subgroups of the related variables (including surgery and scope of regional LN). After including all the clinicopathological variables in the logistic regression model, each patient’s propensity score was calculated, and 1:1 pair matching was performed by greedy nearest neighbor matching with the threshold of standardized difference set as 0.1. All statistical analyses were performed using R version 4.0.2 software. A two-sided p value <0.05 was considered statistically significant.

## Results

### Population characteristics

As shown in **Table 1**, a total of 238 patients diagnosed with stage I SP were extracted from the SEER database. Of them, 182 (76.5%) were diagnosed with a second primary lung adenocarcinoma, and 56 (23.5%) were diagnosed with a second primary lung SCC. For the SP patients, 152 (63.9%) underwent sublobectomy, and 83 (34.9%) underwent ≥4 regional LN dissections. The median latency from the diagnosis of the FP to that of the SP was 38 months (interquartile range (IQR): 23-59 months) (**Table 1**).

**Table 1:** Clinicopathological characteristics of stage IA SP patients and competing risk model investigating the risk factors of their LCSM.

Variables	Distribution N(%)	Univariate analysis		Multivariate analysis	
		SHR (95% CI)	P	SHR (95% CI)	P
Overall	238				
Age at diagnosis (years)	71 (66-77)	1.00 (0.98-1.03)	0.8		
Sex					
Female	153 (64.3)	Reference		Reference	
Male	85 (35.7)	1.76 (1.09-2.83)	0.02	1.61 (0.96-2.71)	0.073
Race/Ethnicity					
NHW	197 (82.8)	Reference			
NHB	18 (7.6)	1.55 (0.65-3.68)	0.32		
Hispanic	9 (3.8)	0.41 (0.06-2.73)	0.35		
NHAPI	14 (5.9)	0.23 (0.03-1.74)	0.16		
Marital status					
Married	133 (55.9)	Reference			
Unmarried	105 (44.1)	0.86 (0.53-1.39)	0.53		
Tumor site of FP					
Upper lobe	142 (59.7)	Reference			
Middle lobe	14 (5.9)	2.10 (0.92-4.82)	0.08		
Lower lobe	82 (34.5)	1.22 (0.73-2.05)	0.44		
Tumor site of SP					
Upper lobe	136 (57.1)	Reference			
Middle lobe	21 (8.8)	0.92 (0.42-2.00)	0.83		
Lower lobe	81 (34.0)	0.84 (0.50-1.42)	0.51		
Tumor laterality of FP					
Center	105 (44.1)	Reference			
Right	133 (55.9)	1.06 (0.65-1.71)	0.82		
Tumor laterality of SP					
Center	106 (44.5)	Reference			
Right	132 (55.5)	1.04 (0.65-1.68)	0.87		
Grade of FP					
I	48 (20.2)	Reference			
II	109 (45.8)	0.97 (0.50-1.90)	0.94		
III/IV	61 (25.6)	1.44 (0.72-2.88)	0.31		
Unknown	20 (8.4)	1.15 (0.46-2.87)	0.77		
Grade of SP					
I	58 (24.4)	Reference			
II	118 (49.6)	1.60 (0.80-3.21)	0.19		
III/IV	45 (18.9)	2.59 (1.21-5.57)	0.015		
Unknown	17 (7.1)	2.12 (0.83-5.41)	0.12		
Surgery of FP					
Sublobectomy	48 (20.2)	Reference			
Lobectomy	190 (79.8)	0.93 (0.52-1.66)	0.8		
Surgery of SP					
Sublobectomy	152 (63.9)	Reference			
Lobectomy	86 (36.1)	0.71 (0.42-1.20)	0.2		
Scope of regional LN of FP					
None	24 (10.1)	Reference			
1~3	39 (16.4)	0.93 (0.37-2.34)	0.88		
≥4	175 (73.5)	0.80 (0.39-1.65)	0.55		
Scope of regional LN of SP					
None	98 (41.2)	Reference			
1~3	57 (23.9)	0.98 (0.58-1.66)	0.93	0.72 (0.40-1.29)	0.27
≥4	83 (34.9)	0.30 (0.15-0.61)	P<0.0001	0.26 (0.12-0.52)	0.0002
AJCC stage of FP					
IA1	30 (12.6)	Reference		Reference	
IA2	97 (40.8)	3.85 (1.19-12.44)	0.024	3.36 (1.07-10.56)	0.038
IA3	70 (29.4)	2.63 (0.77-9.00)	0.12	2.51 (0.76-8.34)	0.13
IB	41 (17.2)	5.46 (1.63-18.26)	0.006	4.74 (1.41-15.94)	0.012
AJCC stage of SP					
IA1	58 (24.4)	Reference		Reference	
IA2	127 (53.4)	1.40 (0.72-2.74)	0.32	1.40 (0.70-2.82)	0.34
IA3	44 (18.5)	2.10 (0.97-4.55)	0.061	1.78 (0.80-3.94)	0.16
IB	9 (3.8)	6.32 (2.58-15.48)	P<0.0001	4.28 (1.46-12.53)	0.008
Pathology of FP					
Adenocarcinoma	183 (76.9)	Reference			
Squamous cell cancer	55 (23.1)	1.20 (0.70-2.05)	0.51		
Pathology of FP					
Adenocarcinoma	182 (76.5)	Reference	0.15		
Squamous cell cancer	56 (23.5)	0.96 (0.54-1.72)	0.9		
Latency (months)	38 (23-59)	0.99 (0.98-1.00)	0.22		

AJCC, American Joint Committee on Cancer; LCSM, lung cancer-specific morality; FP, first primary non-small cell lung cancer; LN, lymph node; NHAPI, non-Hispanic Asian/Pacific Islander; NHB, non-Hispanic black; NHW, non-Hispanic white; SHR, subdistribution hazard ratios; SP, second primary non-small cell lung cancer.

**Figure 2:**
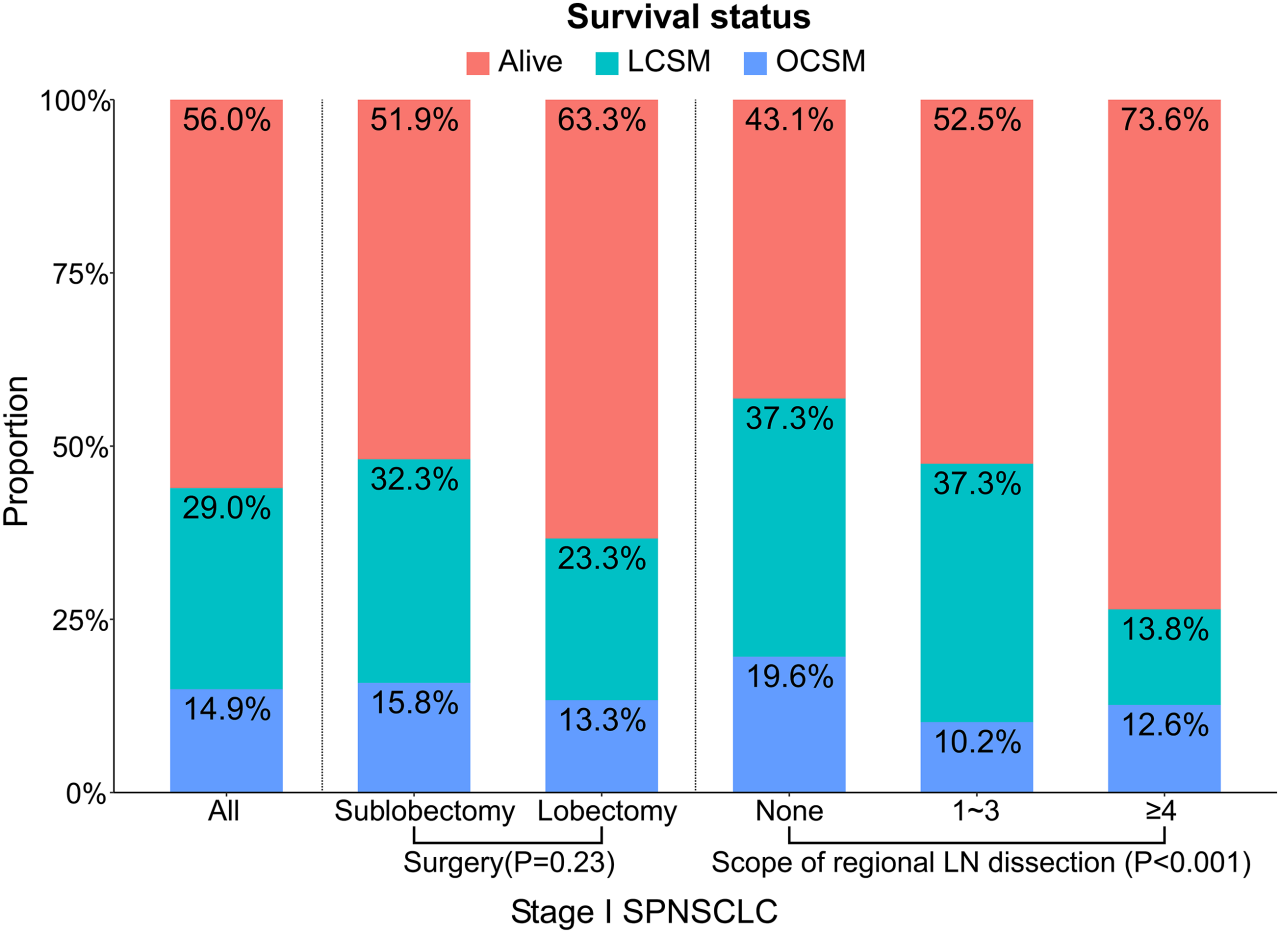
Proportions of different survival outcomes by the related variables (surgery and scope of regional LN dissection) in stage I SP patients. A total of three causes of death were investigated: alive, LCSM, OCSM. The statistical differences were tested using chi-squared tests. LCSM, lung cancer-specific morality; LN, lymph node; OCSM, other cause specific mortality; SP, second primary non-small cell lung cancer.

In addition, we found that the SP patients whose tumor site was located in the upper lobe, the SP patients without regional LN dissection, and the stage IA SP patients all had a higher proportion of sublobectomy (**Table 2**), and the SP patients who underwent lobectomy had a higher proportion of ≥4 regional LN dissections (**Table 3**). A total of 72 (29.0%) patients died of lung cancer, and 37 (14.9%) died of other causes (**Figure 2**). In addition, the SP patients who underwent ≥4 regional LN dissections had the highest proportion of patients alive, while the SP patients without regional LN dissections had the lowest proportion (**Figure 2**).

**Table 2:** Baseline characteristics between the stage I SP patients undergoing sublobectomy and patients undergoing lobectomy before and after propensity score matching.

Variables	Before propensity score matching			After propensity score matching		
	sublobectomy	Lobectomy	P	sublobectomy	Lobectomy	P
n	152	86		47	47	
Age (years)	72 (66-78)	71 (67-76)	0.625	71 (64-80)	71 (67-76)	0.785
Sex			0.732			0.828
Female	96 (63.2)	57 (66.3)		30 (63.8)	32 (68.1)	
Male	56 (36.8)	29 (33.7)		17 (36.2)	15 (31.9)	
Race			0.688			0.951
NHW	123 (80.9)	74 (86.0)		39 (83.0)	38 (80.9)	
NHB	13 (8.6)	5 (5.8)		4 (8.5)	4 (8.5)	
Hispanic	7 (4.6)	2 (2.3)		1 (2.1)	2 (4.3)	
NHAPI	9 (5.9)	5 (5.8)		3 (6.4)	3 (6.4)	
Marital status			1			1
Married	85 (55.9)	48 (55.8)		26 (55.3)	25 (53.2)	
Unmarried	67 (44.1)	38 (44.2)		21 (44.7)	22 (46.8)	
Tumor site of FP			0.184			0.839
Upper lobe	90 (59.2)	52 (60.5)		29 (61.7)	30 (63.8)	
Middle lobe	6 (3.9)	8 (9.3)		2 (4.3)	1 (2.1)	
Lower lobe	56 (36.8)	26 (30.2)		16 (34.0)	16 (34.0)	
Tumor site of SP			0.003			0.676
Upper lobe	98 (64.5)	38 (44.2)		26 (55.3)	22 (46.8)	
Middle lobe	8 (5.3)	13 (15.1)		5 (10.6)	7 (14.9)	
Lower lobe	46 (30.3)	35 (40.7)		16 (34.0)	18 (38.3)	
Tumor laterality of FP			0.879			0.837
Center	66 (43.4)	39 (45.3)		22 (46.8)	24 (51.1)	
Right	86 (56.6)	47 (54.7)		25 (53.2)	23 (48.9)	
Tumor laterality of SP			0.447			0.675
Center	71 (46.7)	35 (40.7)		21 (44.7)	18 (38.3)	
Right	81 (53.3)	51 (59.3)		26 (55.3)	29 (61.7)	
Grade of FP			0.937			0.795
I	31 (20.4)	17 (19.8)		12 (25.5)	12 (25.5)	
II	69 (45.4)	40 (46.5)		19 (40.4)	18 (38.3)	
III	38 (25.0)	23 (26.7)		12 (25.5)	15 (31.9)	
Unknown	14 (9.2)	6 (7.0)		4 (8.5)	2 (4.3)	
Grade of SP			0.103			0.935
I	37 (24.3)	21 (24.4)		10 (21.3)	12 (25.5)	
II	83 (54.6)	35 (40.7)		26 (55.3)	23 (48.9)	
III	23 (15.1)	22 (25.6)		8 (17.0)	9 (19.1)	
Unknown	9 (5.9)	8 (9.3)		3 (6.4)	3 (6.4)	
Surgery of FP			1			1
Sublobectomy	31 (20.4)	17 (19.8)		9 (19.1)	10 (21.3)	
Lobectomy	121 (79.6)	69 (80.2)		38 (80.9)	37 (78.7)	
Scope of regional LN of FP			0.112			0.876
None	20 (13.2)	4 (4.7)		3 (6.4)	4 (8.5)	
1~3	24 (15.8)	15 (17.4)		7 (14.9)	8 (17.0)	
≥4	108 (71.1)	67 (77.9)		37 (78.7)	35 (74.5)	
Scope of regional LN of SP			<0.001			0.51
None	92 (60.5)	6 (7.0)		10 (21.3)	6 (12.8)	
1~3	35 (23.0)	22 (25.6)		16 (34.0)	16 (34.0)	
≥4	25 (16.4)	58 (67.4)		21 (44.7)	25 (53.2)	
AJCC stage of FP			0.019			0.812
IA1	24 (15.8)	6 (7.0)		4 (8.5)	5 (10.6)	
IA2	53 (34.9)	44 (51.2)		18 (38.3)	21 (44.7)	
IA3	51 (33.6)	19 (22.1)		15 (31.9)	11 (23.4)	
IB	24 (15.8)	17 (19.8)		10 (21.3)	10 (21.3)	
AJCC stage of SP			<0.001			0.97
IA1	49 (32.2)	9 (10.5)		9 (19.1)	9 (19.1)	
IA2	79 (52.0)	48 (55.8)		24 (51.1)	24 (51.1)	
IA3	22 (14.5)	22 (25.6)		12 (25.5)	11 (23.4)	
IB	2 (1.3)	7 (8.1)		2 (4.3)	3 (6.4)	
Pathology of FP			0.841			1
Adenocarcinoma	118 (77.6)	65 (75.6)		34 (72.3)	33 (70.2)	
Squamous cell cancer	34 (22.4)	21 (24.4)		13 (27.7)	14 (29.8)	
Pathology of FP			0.687			1
Adenocarcinoma	118 (77.6)	64 (74.4)		33 (70.2)	33 (70.2)	
Squamous cell cancer	34 (22.4)	22 (25.6)		14 (29.8)	14 (29.8)	
Latency (months)	38 (21-61)	39 (26-56)	0.816	42 (21-65)	39 (29-58)	0.871

AJCC, American Joint Committee on Cancer; FP, first primary non-small cell lung cancer; LN, lymph node; NHAPI, non-Hispanic Asian/Pacific Islander; NHB, non-Hispanic black; NHW, non-Hispanic white; SHR, subdistribution hazard ratios; SP, second primary non-small cell lung cancer.

**Table 3:** Baseline characteristics between the stage I SP patients undergoing 1~3 regional LN dissection and patients undergoing ≥4 regional LN dissection before and after propensity score matching.

Variables	Before propensity score matching			After propensity score matching		
	1~3 regional LN dissection	≥4 regional LN dissection	P	1~3 regional LN dissection	≥4 regional LN dissection	P
n	57	83		35	35	
Age (years)	72 (69-77)	70 (65-75)	0.099	72 (67-77)	71 (67-76)	0.76
Sex			0.925			0.623
Female	37 (64.9)	52 (62.7)		20 (57.1)	23 (65.7)	
Male	20 (35.1)	31 (37.3)		15 (42.9)	12 (34.3)	
Race			0.379			0.558
NHW	50 (87.7)	69 (83.1)		30 (85.7)	28 (80.0)	
NHB	4 (7.0)	10 (12.0)		3 (8.6)	3 (8.6)	
Hispanic	0 (0.0)	2 (2.4)		0 (0.0)	2 (5.7)	
NHAPI	3 (5.3)	2 (2.4)		2 (5.7)	2 (5.7)	
Marital status			0.596			0.464
Married	35 (61.4)	46 (55.4)		23 (65.7)	19 (54.3)	
Unmarried	22 (38.6)	37 (44.6)		12 (34.3)	16 (45.7)	
Tumor site of FP			0.464			0.791
Upper lobe	35 (61.4)	51 (61.4)		21 (60.0)	23 (65.7)	
Middle lobe	2 (3.5)	7 (8.4)		2 (5.7)	1 (2.9)	
Lower lobe	20 (35.1)	25 (30.1)		12 (34.3)	11 (31.4)	
Tumor site of SP			0.867			1
Upper lobe	28 (49.1)	44 (53.0)		17 (48.6)	17 (48.6)	
Middle lobe	6 (10.5)	7 (8.4)		5 (14.3)	5 (14.3)	
Lower lobe	23 (40.4)	32 (38.6)		13 (37.1)	13 (37.1)	
Tumor laterality of FP			0.618			1
Center	28 (49.1)	36 (43.4)		16 (45.7)	16 (45.7)	
Right	29 (50.9)	47 (56.6)		19 (54.3)	19 (54.3)	
Tumor laterality of SP			0.419			0.807
Center	20 (35.1)	36 (43.4)		13 (37.1)	15 (42.9)	
Right	37 (64.9)	47 (56.6)		22 (62.9)	20 (57.1)	
Grade of FP			0.245			0.992
I	9 (15.8)	21 (25.3)		7 (20.0)	8 (22.9)	
II	26 (45.6)	39 (47.0)		16 (45.7)	15 (42.9)	
III	16 (28.1)	20 (24.1)		10 (28.6)	10 (28.6)	
Unknown	6 (10.5)	3 (3.6)		2 (5.7)	2 (5.7)	
Grade of SP			0.536			0.779
I	11 (19.3)	19 (22.9)		6 (17.1)	5 (14.3)	
II	28 (49.1)	42 (50.6)		18 (51.4)	22 (62.9)	
III	16 (28.1)	16 (19.3)		9 (25.7)	6 (17.1)	
Unknown	2 (3.5)	6 (7.2)		2 (5.7)	2 (5.7)	
Surgery of FP			0.43			1
Sublobectomy	12 (21.1)	12 (14.5)		5 (14.3)	6 (17.1)	
Lobectomy	45 (78.9)	71 (85.5)		30 (85.7)	29 (82.9)	
Surgery of SP			<0.001			0.63
Sublobectomy	35 (61.4)	25 (30.1)		17 (48.6)	14 (40.0)	
Lobectomy	22 (38.6)	58 (69.9)		18 (51.4)	21 (60.0)	
Scope of regional LN of FP			0.61			0.839
None	5 (8.8)	5 (6.0)		2 (5.7)	1 (2.9)	
1~3	10 (17.5)	11 (13.3)		5 (14.3)	5 (14.3)	
≥4	42 (73.7)	67 (80.7)		28 (80.0)	29 (82.9)	
AJCC stage of FP			0.944			0.959
IA1	7 (12.3)	8 (9.6)		5 (14.3)	5 (14.3)	
IA2	21 (36.8)	34 (41.0)		13 (37.1)	12 (34.3)	
IA3	17 (29.8)	24 (28.9)		9 (25.7)	11 (31.4)	
IB	12 (21.1)	17 (20.5)		8 (22.9)	7 (20.0)	
AJCC stage of SP			0.153			0.981
IA1	5 (8.8)	18 (21.7)		4 (11.4)	5 (14.3)	
IA2	36 (63.2)	47 (56.6)		21 (60.0)	21 (60.0)	
IA3	11 (19.3)	15 (18.1)		8 (22.9)	7 (20.0)	
IB	5 (8.8)	3 (3.6)		2 (5.7)	2 (5.7)	
Pathology of FP			1			0.784
Adenocarcinoma	43 (75.4)	62 (74.7)		25 (71.4)	27 (77.1)	
Squamous cell cancer	14 (24.6)	21 (25.3)		10 (28.6)	8 (22.9)	
Pathology of FP			1			0.591
Adenocarcinoma	42 (73.7)	60 (72.3)		24 (68.6)	27 (77.1)	
Squamous cell cancer	15 (26.3)	23 (27.7)		11 (31.4)	8 (22.9)	
Latency (months)	33 (17-52)	46 (31-62)	0.007	33 (19-49)	46 (29-57)	0.11

AJCC, American Joint Committee on Cancer; FP, first primary non-small cell lung cancer; LN, lymph node; NHAPI, non-Hispanic Asian/Pacific Islander; NHB, non-Hispanic black; NHW, non-Hispanic white; SHR, subdistribution hazard ratios; SP, second primary non-small cell lung cancer.

### Cumulative incidences of LCSM

**Figure 3:**
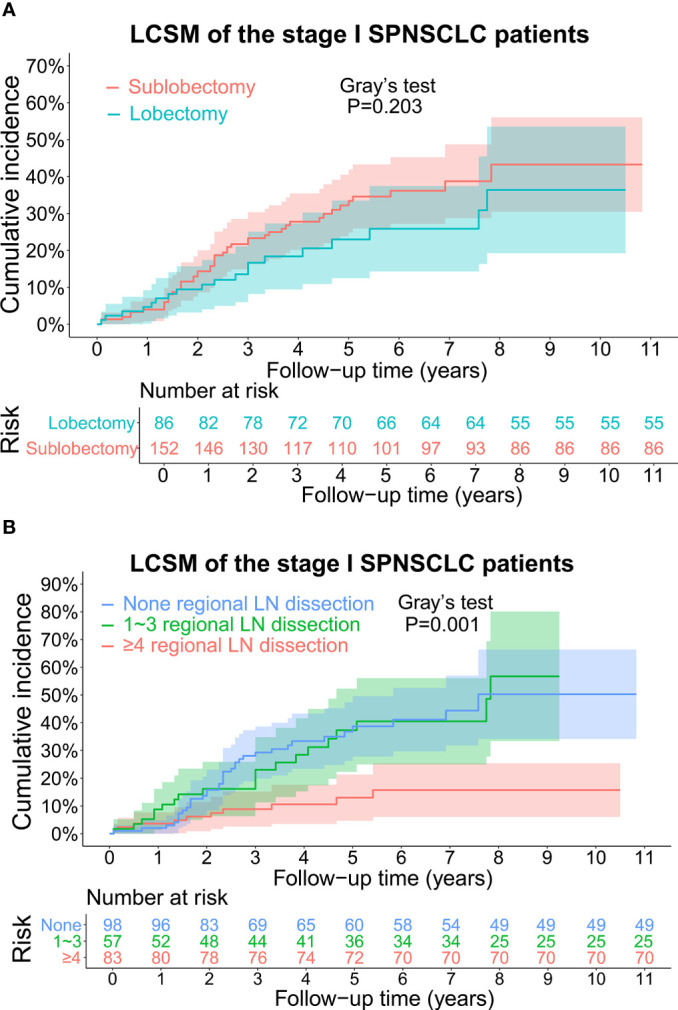
**(A)** CIF curves for estimating the LCSM in the whole stage I SP cohort before propensity score matching, and the differences in CIF between the patients undergoing sublobectomy and patients undergoing lobectomy. The number of patients at risk was listed under the figure, and the shaded bands represented the 95% confidence intervals. P-value of difference in CIF was determined based on Gray’s test. **(B)** CIF curves for estimating the LCSM in the whole stage I SP cohort before propensity score matching, and the differences in CIF among the patients without regional LN dissection, patients undergoing 1~3 regional LN dissection and patients undergoing ≥4 regional LN dissection. The number of patients at risk was listed under the figure, and the shaded bands represented the 95% confidence intervals. P-value of difference in CIF was determined based on Gray’s test. CIF, cumulative incidence function; LCSM, lung cancer-specific morality; LN, lymph node; SP, second primary non-small cell lung cancer.

**Figure 4:**
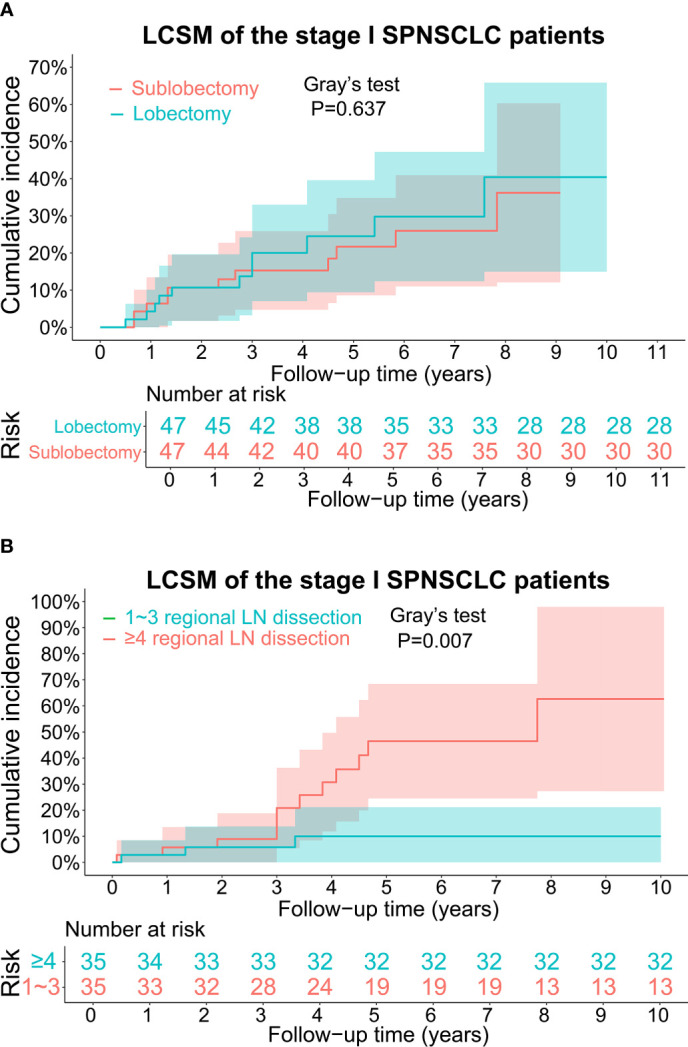
**(A)** CIF curves for estimating the LCSM in the stage I SP cohort after propensity score matching, and the differences in CIF between the patients undergoing sublobectomy and patients undergoing lobectomy. The number of patients at risk was listed under the figure, and the shaded bands represented the 95% confidence intervals. P-value of difference in CIF was determined based on Gray’s test. **(B)** CIF curves for estimating the LCSM in the stage I SP cohort after propensity score matching, and the differences in CIF between the patients undergoing 1~3 regional LN dissection and patients undergoing ≥4 regional LN dissection. The number of patients at risk was listed under the figure, and the shaded bands represented the 95% confidence intervals. P-value of difference in CIF was determined based on Gray’s test. CIF, cumulative incidence function, LCSM, lung cancer-specific morality; LN, lymph node; SP, second primary non-small cell lung cancer.

The CIF curves for estimating the LCSM by different treatment characteristics (surgery and scope of regional LN dissection) of the stage I SP patients are displayed in **Figures 3**, **4**. Overall, the 5-year LCSM rate was 29.8% (CI: 23.1%-36.5%) for the whole cohort. Moreover, the 5-year LCSM rates of the stage I SP patients who underwent sublobectomy and lobectomy were 33.6% (CI: 24.9%-41.9%) and 23.3% (CI: 12.5%-33.5%), respectively. Meanwhile, the 5-year LCSM rates of the patients who underwent 0, 1~3, and ≥4 regional LN dissections were 38.8% (CI: 27.8%-49.6%), 36.8% (CI: 22.3%-52.3%) and 13.3% (CI: 4.7%-21.3%), respectively. For the whole cohort before PSM, we found that the stage I SP patients who underwent lobectomy had a similar cumulative incidence of LCSM to those who underwent sublobectomy (**Figure 3A**), while the patients who underwent ≥4 regional LN dissections had a significantly lower cumulative incidence of LCSM than those who underwent 1~3 regional LN dissections or without LN dissections (**Figure 3B**). The above findings were quite robust even if all clinicopathological variables were matched using PSM (**Figure 4A, B**).

### Univariate and multivariate competing risk models before PSM

**Figure 5:**
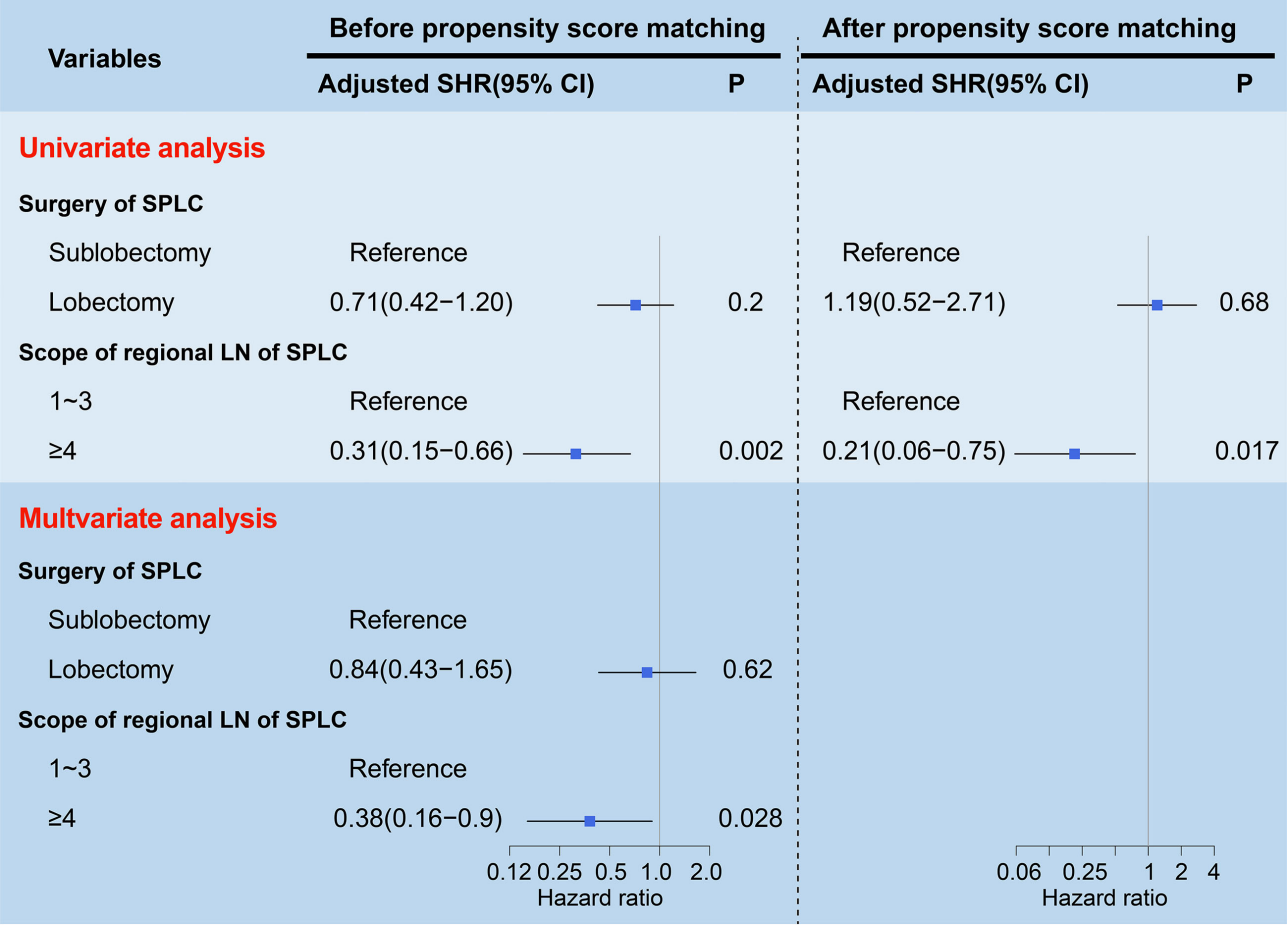
Forest plots visualizing the SHRs and P-values of the predictors (surgery and scope of regional LN) using the univariate and multivariate competing risk models before and after propensity score matching. The gray vertical solid line indicates SHR of 1. LN, lymph node; SP, second primary non-small cell lung cancer; SHR, subdistribution hazard ratio; CI, confidence interval.

To assess the LCSM of stage I SP patients before PSM, both univariate and multivariate competing risk models were used. In the univariate analysis, the patients who underwent lobectomy had a similar incidence of LCSM as those who underwent sublobectomy (SHR, 0.93; 95% CI, 0.52-1.66; P=0.8) (**Table 1**). Regarding the scope of regional LN dissection, the patients who underwent ≥4 regional LN dissections had a significantly lower incidence of LCSM than those without LN dissections (SHR, 0.3; 95% CI, 0.15-0.61; P<0.0001), while the patients who underwent 1~3 regional LN dissections had a similar incidence of LCSM to those without LN dissections (SHR, 0.98; 95% CI, 0.58-1.66; P=0.93) (**Table 1**). In the multivariate analysis, the patients who underwent ≥4 regional LN dissections still had a significantly lower incidence of LCSM than those without LN dissections (SHR, 0.26; 95% CI, 0.12-0.52; P=0.0002) (**Table 1**). Subsequently, we further investigated the survival differences between the patients who underwent ≥4 regional LN dissections and the patients who underwent 1~3 regional LN dissections. As shown in **Figure 5**, regardless of whether determined by the univariate analysis or multivariate analysis, the patients who underwent ≥4 regional LN dissections had a significantly lower LCSM incidence than those who underwent 1~3 regional LN dissections.

### Univariate and multivariate competing risk models after PSM

To further validate the above survival disparities, PSM was used, and all the related clinicopathological variables were matched successfully, with all P > 0.05 (**Tables 2**, **3**). After PSM, we found that surgery was still not a prognostic factor for stage I SP patients (SHR, 1.19; 95% CI, 0.52-2.71; P=0.68) (**Figure 5**), and the patients who underwent ≥4 regional LN dissections had a significantly lower LCSM incidence than those who underwent 1~3 regional LN dissections (SHR, 0.21; 95% CI, 0.06-0.75; P=0.017) (**Figure 5**).

## Discussion

Currently, the optimal treatment strategy for SP patients is still controversial. Some studies revealed that lobectomy was the optimal choice for SP patients (15, 16). However, others insisted that sublobectomy shared a similar survival with lobectomy, and sublobectomy could be perceived as a better surgical method for SP patients (12–14). In this study, we found that stage I SP patients following postoperative stage I FP gained more survival benefits when ≥4 regional LN dissections were performed. However, surgical method (lobectomy and sublobectomy) was not a survival-related factor for these patients. To the best of our knowledge, this is the first study to adopt competing risk models to investigate the optimal treatment strategy for stage I SP patients following postoperative stage I FP.

Recently, two large prospective randomized control trials have been performed to investigate the role of sublobar resection in early-stage lung cancer. One trial exploring the survival disparities between patients who underwent lobectomy and those who underwent sublobar resection for peripheral stage IA NSCLC revealed that sublobar resection was similar to lobectomy in overall survival and was not inferior to lobectomy in terms of disease-free survival (22). The other trial (JCOG0802/WJOG4607L) found that segmentectomy was superior to lobectomy in overall survival and was similar to lobectomy in relapse-free survival for clinical stage IA NSCLC (23). Although these prospective trials have demonstrated the similarity or even superiority of segmentectomy for overall survival versus lobectomy for stage IA NSCLC, they mainly focused on the FP rather than SP. Allowing for the lower incidence of stage I SP patients following the surgery of stage I FP (238 of our study population extracted from over 390,000 NSCLC patients in the SEER database), it may be arduous to carry out one prospective randomized control trial for these patients. Therefore, our findings may, to some extent, play an instructive role in the surgical choice for stage I SP patients following surgery for stage I FP.

In this study, we found that stage I SP patients who underwent ≥4 regional LN dissections had a significantly lower LCSM incidence than those who underwent 1~3 regional LN dissections, which was a similar finding to those from previous studies. Zhang R et al. focused on patients who developed early-stage SP following lobectomy of the initial primary lung cancer and revealed that patients who underwent ≥10 LN examination had a longer OS than those who underwent <10 LN examination (15). In addition, Yan Chen conducted a retrospective study using the SEER database to investigate stage I SP patients and found that the number of LN examinations was correlated with better OS and LCSM in SP patients (18). Based on our findings, we recommend that thoracic surgeons perform ≥4 regional LN dissections for stage I SP patients.

In addition, we discovered that the surgical method (lobectomy and sublobectomy) was not a survival-related factor for stage I SP patients. Similar results have also been found in other studies. Congkuan Song et al. explored the surgical procedure for stage IA SP patients in the SEER database and showed that wedge resection displayed a similar OS and LCSM as lobectomy (13). Another study on early-stage SP after small cell lung cancer (SCLC) also revealed no significant survival difference between patients who underwent sublobar resection and lobectomy (12). In the study by Wang Z et al., the SEER database was used to investigate the surgical choice for SP lesions ≤ 2 cm and demonstrated that there were no significant survival differences among the groups of patients who underwent lobectomy, segmentectomy, and wedge resection (14). Overall, sublobectomy gained a similar survival benefit as lobectomy for stage I SP patients.

The surgical choice for SP is a challenging job for thoracic surgeons, as they should simultaneously take maximal preservation of the residual pulmonary lobe and maximal tumor resection into consideration. Allowing for the damaged pulmonary function caused by the surgical resection of the FP, sublobectomy, which can achieve the relative larger preservation of the residual pulmonary lobe and has a comparable survival to lobectomy, may be more suitable for stage I SP patients. Based on our findings and the above reasons, sublobectomy may be a reasonable choice for thoracic surgeons when performing surgery for stage I SP patients who have undergone surgery for stage I FP. Meanwhile, an adequate resection margin (≥2 cm) is also essential for sublobectomy (3). Additionally, although sublobectomy has been demonstrated as a more preferable surgical method than lobectomy for stage I SP patients, ≥4 regional LN dissections are still indispensable when sublobectomy is performed. Based on the above reasons, we recommend that thoracic surgeons perform sublobectomy in combination with ≥4 regional LN dissections for stage I SP patients.

The major strength of our study is that we only focused on stage I patients (both SP and FP), which could eliminate the major defect from the SEER database, which is that treatment information (such as chemotherapy, targeted therapy, or immunotherapy) sometimes was not available. Undoubtedly, a majority of stage I patients do not require postoperative adjuvant treatments, while stage II, III, IV patients generally should receive postoperative adjuvant treatments. Therefore, the lack of treatment information in the SEER database can significantly influence the prognosis of stage II, III, and IV patients. Additionally, although we only included 238 patients in our study, these patients were screened from over 390,000 NSCLC patients in the SEER database, and the database covered approximately 27.8% of the US population. Allowing for the lower incidence of stage I SP patients following surgery for stage I FP, our patient samples were relatively larger.

Several limitations should be noted. First, this study was a retrospective study, which inevitably led to a selection bias in our results. Therefore, a further prospective study is warranted to validate our findings. Second, several known prognosis-related factors, such as comorbidities, cigarette smoking, and imaging findings, were not available in the SEER database. Third, although we confined our study population to stage I SP patients, it was rational to further conduct a subgroup analysis based on the AJCC stage (IA1, IA2, IA3, IB) of SP to investigate the surgical information. Due to the small size of our study population, it was not suitable to divide them into four groups.

In conclusion, for stage I SP patients following postoperative stage I FP, we demonstrated that ≥4 regional LN dissections were an independent favorable factor. However, surgical method (lobectomy and sublobectomy) was not a prognosis-related factor for these patients. Therefore, if surgeons dissect ≥4 regional LNs for stage I SP patients, patients tend to gain more survival benefits. Additionally, sublobectomy may be a reasonable choice for thoracic surgeons when performing surgery for stage I SP patients who have undergone surgery for FP.

## Data availability statement

Publicly available datasets were analyzed in this study. This data can be found here: https://seer.cancer.gov. Further data are available under request to the corresponding authors.

## Ethics statement

Ethical review and approval was not required for the study on human participants in accordance with the local legislation and institutional requirements. Written informed consent for participation was not required for this study in accordance with the national legislation and the institutional requirements.

## Author contributions

XW, JL and YJ designed and wrote the manuscript; XW, JW, QC, and JL participated in literature search, data acquisition, data analysis, or data interpretation; XW, YJ, and JL contributed to the revision of manuscript. All authors contributed to the article and approved the submitted version.
